# Combination Treatment of Balloon Pulmonary Angioplasty and Direct Oral Anticoagulant in a Patient with Chronic Thromboembolic Pulmonary Hypertension Complicated by Protein S Deficiency

**DOI:** 10.3390/medicina59050909

**Published:** 2023-05-09

**Authors:** Toshihide Izumida, Teruhiko Imamura, Ryuichi Ushijima, Koichiro Kinugawa

**Affiliations:** Second Department of Medicine, University of Toyama, Toyama 930-0194, Japan

**Keywords:** cardiology, pulmonary artery hypertension, hemodynamics, inherited thrombophilia

## Abstract

*Introduction*: Chronic thromboembolic pulmonary hypertension (CTEPH) is a phenotype of pulmonary hypertension due to chronic and multiple organized thrombus. The therapeutic strategy for patients with CTEPH and comorbid protein S deficiency remains unknown due to its rarity. *Case*: We encountered a 49-year-old male patient with CTEPH and concomitant mild protein S deficiency (type III). We could successfully perform balloon pulmonary angioplasty without any major complications, including thromboembolism and bleeding, followed by standard-dose oral anticoagulation therapy instead of warfarin. *Conclusion*: A currently established standard therapeutic strategy for CTEPH, including pulmonary angioplasty, may be safe and effective even in patients with concomitant inherent coagulation abnormalities.

## 1. Introduction

Chronic thromboembolic pulmonary hypertension (CTEPH) is a rare disease characterized by increased pulmonary vascular resistance and ventilatory dysfunction due to mechanical occlusion and microvascular injury from organized thrombus in multiple pulmonary vasculature [1,2].

Protein S deficiency causes venous thromboembolism, including CTEPH, due to an abnormality in the function of downregulation of the thrombin generation [3]. Although protein S deficiency is more common in Asia, it may be much rarer, especially in Europe and America [4], and there are few case reports regarding CTEPH complicated by protein S deficiency [5,6]. The inherited coagulopathy, including protein S deficiency, could theoretically cause massive thrombus and occlude relatively larger pulmonary vessels, with an increased risk of hemodynamic deterioration and reperfusion pulmonary edema after pulmonary angioplasty [7,8]. Given its unique pathophysiology, special attention and specific therapeutic strategy may be required for patients with such diseases.

Here, we presented a patient with CTEPH complicated by protein S deficiency successfully treated with balloon pulmonary angioplasty and discussed the therapeutic strategy in a patient with CTEPH and protein S deficiency.

## 2. Case Presentation

### 2.1. Past History

We present a 49-year-old man with CTEPH and protein S deficiency who was admitted to our hospital for balloon pulmonary angioplasty. The patient had dyspnea on exertion one year ago and was admitted to the previous hospital. Pulmonary perfusion scintigraphy showed a wedge-shaped defect in both lungs. A contrast computed tomography showed thrombi in the bilateral pulmonary arteries. A right heart catheterization revealed 31 mmHg of mean pulmonary artery pressure and 4.5 Wood units of pulmonary vascular resistance. 

Chronic thromboembolic pulmonary hypertension was suspected. A protein S free antigen level was decreased, and protein S antigen activity was 21%, both of which were retested and decreased, indicating type III protein S deficiency (mild protein S deficiency, instead of moderate or severe). Rivaroxaban was started. 

### 2.2. Before Referral

Following 6-month anti-coagulation therapy, mean pulmonary artery pressure decreased to 21 mmHg, and pulmonary vascular resistance was 3.6 Wood units, while residual thrombus in bilateral lungs remained in pulmonary angiography. Pulmonary artery wedge pressure was 14 mmHg. His baseline exercise capacity had been high. Before the development of CTEPH, he could run 20 km without dyspnea but became aware of shortness of breath during a 5-km run following the development of CTEPH. Therefore, he was referred to our institute for further intervention.

### 2.3. On Admission

On admission, he complained of dyspnea on light exertion with WHO functional class II. His body height was 165.6 cm, and his body weight was 60.3 kg. Blood pressure was 127/77 mmHg, pulse rate was 60 bpm, and resting saturation in the room was 97%. Plasma B-type natriuretic peptide level was 29.4 pg/mL.

**Figure 1:**
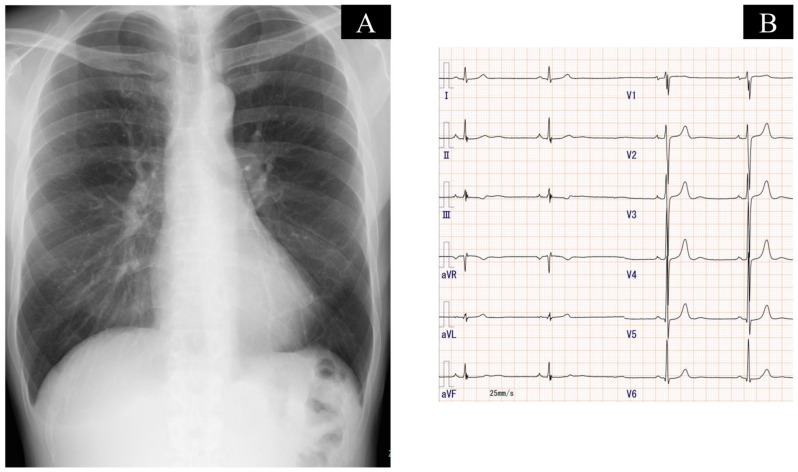
Chest X-ray that shows no enlargement of central pulmonary arteries, cardiomegaly, and pleural effusion (**A**), and electrocardiography that shows sinus bradycardia and left ventricular hypertrophy (**B**).

**Figure 2:**
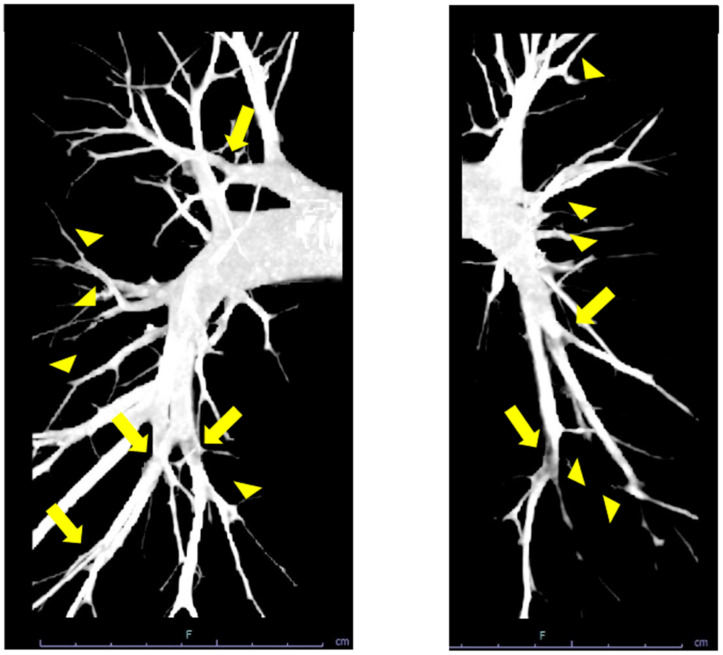
Contrast computed tomography showing web-like strictures (arrows) and abrupt narrowing (arrow heads) in bilateral pulmonary arteries.

Chest X-ray displayed neither enlargement of central pulmonary arteries, cardiomegaly, nor pleural effusion (Figure 1A). Electrocardiography depicted sinus bradycardia and left ventricular hypertrophy without right axis deviation, dominant R wave in V1 lead, and dominant S wave in V5 or V6 lead (Figure 1B). Transthoracic echocardiography showed a left ventricular end-systolic diameter of 43 mm and a left ventricular ejection fraction of 68%. The wall motion of the left ventricle was normal without D-shape. He had mild tricuspid regurgitation with 28 mmHg of estimated pressure gradient. Contrast computed tomography displayed web-like structures and abrupt narrowing in bilateral pulmonary arteries (Figure 2). He underwent the first cardiopulmonary exercising test on a cycle ergometer using a 15-W ramp protocol. He achieved a peak exercise respiratory exchange ratio of 1.06 and a peak VO_2_ of 35.1 mL/kg/min (131% of age-predicted value). The minimum exercise saturation was 92%.

### 2.4. Balloon Pulmonary Angioplasty

**Figure 3:**
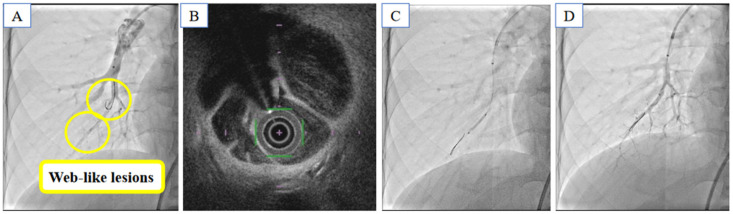
Balloon pulmonary angioplasty for web-like lesions in the right pulmonary artery. Pulmonary angiography and optical frequency domain imaging showed web stenosis lesions (**A**,**B**). The wire was introduced, and the balloon was inflated in both distal and proximal lesions (**C**). Pulmonary angiography showed the rupture of web stenosis in the pulmonary artery (**D**).

**Figure 4:**
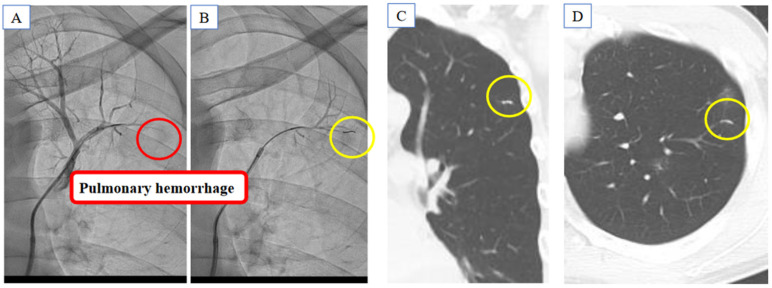
Pulmonary hemorrhage was seen due to wire perforation in the segment A1 + 2 in the left pulmonary artery (**A**), and coil embolization (yellow circle) was performed (**B**–**D**).

Balloon pulmonary angioplasty was performed for lesions in 9 segments in the right pulmonary artery and 6 segments in the left pulmonary artery (Figure 3). Pulmonary hemorrhage was noted due to wire perforation of the segment A1 + 2 in the left pulmonary artery, and coil embolization was performed (Figure 4). After a second session of balloon pulmonary angioplasty, mean pulmonary artery pressure decreased from 21 mmHg to 18 mmHg.

### 2.5. Following Procedure

Prior to discharge, he underwent a second cardiopulmonary exercise test to reassess his exercise tolerance. He achieved a peak exercise respiratory exchange ratio of 1.16 and a peak VO_2_ of 38.0 mL/kg/min (142% of age-predicted value). The minimum exercise during exercise improved from 92% to 95%. He was discharged without initiation of pulmonary arterial hypertension medication. After the index discharge, he could run more than 5 km without dyspnea.

## 3. Discussion

### 3.1. Therapeutic Strategy for CTEPH and Protein S Deficiency

The essential elements in the management of CTEPH are early recognition of the disease and combined treatment with pulmonary endarterectomy, balloon pulmonary angioplasty, and medical therapy [2]. If the inherited thrombophilia is complicated, including protein S deficiency, the main trunk of the pulmonary artery might be injured by massive thrombus formation accompanying hemodynamic deterioration, and the risk of reperfusion pulmonary edema following interventions might increase [7,8].

There are no studies discussing therapeutic strategies for such conditions, except for some case reports. Ando et al. reported that pulmonary endarterectomy was effective in eight CTEPH patients with inherited thrombophilia [5]. Akbayrak H et al. reported a case of successful pulmonary endarterectomy in a patient with CTEPH associated with protein C and protein S deficiency [6]. We performed balloon pulmonary angioplasty with no major problems, such as pulmonary edema and thrombotic events, except for a trivial procedure-related pulmonary hemorrhage. The standard procedure might be safe and effective in patients with CTEPH and protein S deficiency, at least when their severity is mild, as in our patient.

In terms of medical intervention for protein S deficiency, we preferred direct oral anticoagulants to warfarin for several reasons: Warfarin induces transient hyper-coagulopathy due to the blockade of production of protein S, leading to warfarin-induced skin necrosis in patients with inherited coagulopathy [9]. In patients with deep vein thrombosis and inherited thrombosis, a randomized control trial demonstrated a non-inferiority of the direct oral anticoagulant with warfarin in terms of the incidence of thrombus-related deaths and pulmonary emboli [10]. Furthermore, warfarin decreases protein S activity, and transient drug termination is required for each re-assessment of protein S activity during the therapeutic period [3]. We preferred standard doses, but the optimal dose of oral anticoagulants remains a future concern.

### 3.2. Angioplasty to Improve Exercise Tolerance

Given the recent improvement in survival after angioplasty in CTEPH, the next concern is the patient’s quality of life [11]. Recent studies have clarified that angioplasty improves exercise capacity even in CTEPH patients without pulmonary hypertension (recently referred to as chronic thromboembolic disease) [2,12]. Nevertheless, the impact of angioplasty in CTEPH patients with “supernormal” hemodynamics remains controversial [13].

Our patient had persistent subjective symptoms despite objectively normal hemodynamics. Both symptoms and hemodynamics further improved following successful angioplasty in our patient. Further studies are warranted to validate the impact of angioplasty on patients’ subjective symptoms, including quality of life and exercise capacity, rather than hemodynamics. Optimal patient selection and appropriate therapeutic targets beyond hemodynamics also remain the next concern.

In addition to angioplasty, medical therapy, including riociguat, may improve exercise tolerance, and combination therapy is expected [14].

## 4. Conclusions

The current established standard therapeutic strategy for CTEPH, including pulmonary angioplasty, might be safe and effective, even in patients with concomitant inherent coagulation abnormalities.

## Data Availability

Data are available from the corresponding author upon reasonable request.
